# Disentangling Biodiversity and Climatic Determinants of Wood Production

**DOI:** 10.1371/journal.pone.0053530

**Published:** 2013-02-20

**Authors:** Montserrat Vilà, Amparo Carrillo-Gavilán, Jordi Vayreda, Harald Bugmann, Jonas Fridman, Wojciech Grodzki, Josephine Haase, Georges Kunstler, MartJan Schelhaas, Antoni Trasobares

**Affiliations:** 1 Estación Biológica de Doñana (EBD-CSIC), Sevilla, Spain; 2 Centre de Recerca Ecològica i Aplicacions Forestals (CREAF), Edifici C, Campus de Bellaterra (UAB), Barcelona, Spain; 3 Forest Ecology, Institute of Terrestrial Ecosystems, Department of Environmental Sciences, ETH Zurich, Zurich, Switzerland; 4 Swedish National Forest Inventory, Department of Forest Resource Management, Swedish University of Agricultural Sciences, Umea, Sweden; 5 Forest Research Institute, Department of Forest Management in Mountain Regions, Kraków, Poland; 6 Faculty of Biology, Geobotany, Albert-Ludwigs University of Freiburg, Freiburg, Germany; 7 Ecosystem Management, Institute of Terrestrial Ecosystems, Department of Environmental Sciences, ETH Zurich, Zurich, Switzerland; 8 Irstea, UR Ecosystèmes Montagnards, ST-Martin-D'heres, France; 9 Alterra, Wageningen University and Research Centre, Wageningen, The Netherlands; The Pennsylvania State University, United States of America

## Abstract

**Background:**

Despite empirical support for an increase in ecosystem productivity with species diversity in synthetic systems, there is ample evidence that this relationship is dependent on environmental characteristics, especially in structurally more complex natural systems. Empirical support for this relationship in forests is urgently needed, as these ecosystems play an important role in carbon sequestration.

**Methodology/Principal Findings:**

We tested whether tree wood production is positively related to tree species richness while controlling for climatic factors, by analyzing 55265 forest inventory plots in 11 forest types across five European countries. On average, wood production was 24% higher in mixed than in monospecific forests. Taken alone, wood production was enhanced with increasing tree species richness in almost all forest types. In some forests, wood production was also greater with increasing numbers of tree types. Structural Equation Modeling indicated that the increase in wood production with tree species richness was largely mediated by a positive association between stand basal area and tree species richness. Mean annual temperature and mean annual precipitation affected wood production and species richness directly. However, the direction and magnitude of the influence of climatic variables on wood production and species richness was not consistent, and vary dependent on forest type.

**Conclusions:**

Our analysis is the first to find a local scale positive relationship between tree species richness and tree wood production occurring across a continent. Our results strongly support incorporating the role of biodiversity in management and policy plans for forest carbon sequestration.

## Introduction

The rapid loss of biodiversity in the last century has opened a debate on the consequences for ecosystem functioning. Therefore, understanding whether there is a relationship between species diversity and ecosystem processes is a key priority in the face of major global changes [1], [2], [3]. One of the most explored relationships has been between plant species richness and productivity, a process determining ecosystem carbon (C) pools and fluxes, and closely linked to ecosystem C sequestration [4], [5]. Most studies conducting manipulative experiments have found a positive effect of species richness on productivity [2], [6]. However, as these experiments are conducted in simplistic settings (e.g. even-aged species with short life cycles), there is controversy whether this effect holds in structurally more complex natural systems.

Forest ecosystems are major terrestrial C sinks, with a larger capacity to remove atmospheric C than previously thought [7]. Wood production is one of the main components of atmospheric C sequestration in the biosphere, with a high spatial variation depending on biotic, environmental and management factors [8]. Given the global interest in mitigating the consequences of greenhouse gases in the atmosphere, and the need for biodiversity conservation, it is necessary to determine to what extent wood production is reduced by the loss of tree species diversity, and to pinpoint differences among forest types [4], [9], [10].

The tree species richness-productivity relationship has been investigated in forests by analyzing forest inventory data [11], [12], [13], [14], experimentally by manipulating tree species diversity in plantations [15], [16], [17], [18], [19] and by simulation modeling [20], [21]. Studies based on forest inventory data have the potential for testing whether there is a positive relationship between tree species richness and wood production in the “real world”. However, such studies must control for the spatial heterogeneity of forest structure and confounding environmental factors such as climate [14]. To date, most studies have been conducted within certain climatic regions and for particular monospecific-mixed assemblages (e.g. [22], [23]), while only few have encompassed large environmental gradients including a variety of forest types (cf. [12], [13], [14], [24]).

By using unpublished data from more than 55000 forest inventory plots across Europe, we constructed Structural Equation Models (SEM) to test for the direct and indirect dependence of wood production on tree species richness while accounting for stand structure and climatic factors. The hypotheses tested were:

1. Wood production is positively and directly related to tree species richness.
2. Wood production is positively and directly related to the richness of functional tree types. The rationale for this is that tree functional types represent main differences in tree life-history and resource use. Therefore, ecosystem functioning might be as related to tree type richness as to species richness *per se* [4].
3. Wood production indirectly increases with tree species richness through a positive effect of tree species richness on tree stand basal area. Our rationale for this hypothesis is that because most European forests have been largely managed in the past, they are predominantly early successional secondary forests (i.e. young forests) that have not reached maximum size and still accumulate carbon [25]. Under these circumstances, stand basal area is expected to be positively associated with local tree species richness [22], [26].
4. The positive association between wood production and tree species richness still remains when controlling for differences in climatic conditions. Our prediction is that mean annual precipitation and mean annual temperature have a parallel influence on both wood production and tree species richness [27].

## Materials and Methods

### Database and selected variables

We collated forest inventory datasets from five European countries (France, the Netherlands, Spain, Sweden and Switzerland) on the basis of their quality and accurate evaluation of aboveground wood production. With the exception of France, inventories have been conducted in permanent plots surveyed from 1983 to 2009. We selected pairs of contiguous surveys ranging from 5 to 13.5 year periods. The French forest inventory is based on temporary plots where the volume growth of each tree over the last five years is estimated retrospectively based on radial and height growth measurements. In France, only data for the Alps and the Jura Mountains (southeast France) were available for this analysis. The basic criteria of plot selection were the lack of human intervention during contiguous surveys, and that all trees in the plot had been measured above a diameter at breast height (DBH) threshold (Table S1). Detailed information on inventory data for each European country is summarized in Table S1 [28].

For each selected plot, we assigned the forest type according to the European Environmental Agency classification (EEA 2006). In total, our dataset included 55265 plots of 11 European forest types (Table 1). Tree species were also classified into four coarse tree functional types: evergreen conifers, deciduous conifers, evergreen broadleaved -sclerophyllous- and deciduous broadleaved trees). For each plot, tree species richness and tree type richness were calculated. The number of tree species per plot (tree species richness) ranged from one to ten. On average, 49.39% of the plots were mixed with two and three tree species mixtures being the most common (28.21% and 13.56%, respectively). Less than 1% of the plots had more than six tree species. Boreal, hemiboreal and broadleaved evergreen forest plots had a maximum of five tree species. The highest tree species richness was found in mesophytic deciduous forests (ten species per plot), and in floodplain forests and exotic plantations (nine species per plot). The number of tree types (hereafter tree type richness) ranged from one to three. Most commonly, plots had only one tree type (68.76%). Plots with three tree types were rare (2.32%). Table 1 provides information on the number of monospecific and mixed plots for each forest type.

**Table 1:** Main characteristics of forest plots.

	Acidophilous oak	Alpine coniferous	Beech	Boreal and hemiboreal	Broadleaved evergreen	Coniferous Mediterranean	Exotic plantations	Floodplain	Mesophytic deciduous	Non-riverine pioneer	Thremophilous deciduous
**Number** **of plots** **(mono/** **mixed)**	14/105	5655/7064	563/1826	515/2504	7114/3285	9627/4294	1254/2358	43/162	2381/4167	110/644	692/888
**Countries** *	NL	SPA/FRA/SWI	FRA/NL/ SPA/SWI	NL/SWE/SWI	FRA/SPA	FRA/SPA	FRA/NL/SPA/ SWE/SWI	FRA/NL/SPA/SW	FRA/NL/SPA/SWI	FRA/NL/SPA/ SWE/SWI	SPA/SWI
**Annual** **precipitation** **(mm)**	811.8±28.7	910.3±302	1263±335.3	646.5±110.2	669.3±157.7	586.2±217.1	1262±409.5	1040.3±440.3	1052.2±291.4	831±336	726.2±214.9
**Annual** **temperature** **(°C)**	9.52±0.19	9.3±2.1	8.7±1.4	3.6±3	14±2.2	13.5±1.9	10.4±3.4	11.2±2.3	10.6±1.6	6±4.3	12±1.8
**Dominant** **species**	*Betula pendula, Quercus robur*	*Abies alba, Pinus nigra, P. sylvestris*	*Fagus sylvatica*	*Picea abies, P. sylvestris*	*Quercus ilex, Q. suber*	*P. halepensis, P. pinaster, P. pinea*	*Eucalyptus globules, Picea abies, P. radiata*	*Alnus glutinosa, Populus nigra, Salix spp.*	*Q. petraea, Q. pubescens, Q. pyrenaica*	*Betula spp., Populus alba, Populus nigra, Populus tremula*	*Fraxinus angustifolia, Q. faginea*
**Stand** **basal área** **(m^2^/ha)**	21.4±7.4	21.7±14	27.3±13.1	22±11	7.4±5.7	12.7±10.2	26.2±17.1	16.2±10.9	17.3±11.4	17.5±11.4	9.4±7.8
**Plot** **size (ha)**	0.05±0	0.08±0.06	0.09±0.07	0.03±0	0.12±0.07	0.1±0.06	0.07±0.06	0.12±0.07	0.07±0.06	0.05±0.04	0.1±0.07
**Tree species** **richness/plot**	3.3±1.3	1.8±1	2.6±1.3	2.2±0.7	1.4±0.6	1.4±0.7	2.2±1.3	3±1.8	2.3±1.4	2.6±1.2	1.8±0.9
**Tree type** **richness/plot**	1.5±0.5	1.4±0.5	1.4±0.5	1.7±0.5	1.3±0.5	1.2±0.5	1.5±0.5	1.3±0.5	1.3±0.5	1.6±0.5	1.5±0.6
**Wood** **production** **(t/ha/yr)**	2±2.3	2±1.7	3.1±2	2.5±2.1	0.49±0.47	1.2±1.3	4.4±4	2.6±2.1	2±1.67	2.6±2.1	0.7±0.7

* Country nomenclature: France (FRA), The Netherlands (NL), Spain (SPA), Sweden (SWE) and Switzerland (SWI). Values indicate means (±SD).

plot as follows:

In inventories based on permanent plots, for each living tree with a minimum DBH of 4–12 cm depending on the country (Table S1), the species identity was noted and tree volume (*V*) was calculated with species-specific functions of *DBH* and *H* fitted on field data from the respective countries as:
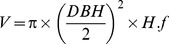
where *f* is the form factor of each species. Wood biomass (*B*) was estimated as:



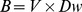
where *Dw* is tree wood density of the species.

The annual increase in aboveground biomass of surviving trees *s* (BG_s_) was measured as:
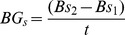



where *Bs_1_* is the biomass of a surviving tree measured in the first survey (1) and still alive in the second survey (2) and *t* is the time elapsed between the two surveys.

Aboveground wood production per plot (*WP*) was estimated as:
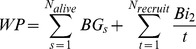
where *N_alive_* is the number of surviving trees in the plot and *BG_s_* their respective annual increase in aboveground biomass. *N_recruit_* is the number of recruited trees during the two contiguous surveys (i.e. trees reaching the minimum DBH of 4–12 cm to be included in the survey), *Bi_2_* is their aboveground biomass and *t* is the time elapsed between the two surveys.

In France, *BG_s_* were computed with an estimation of volume growth over the last five years for each tree alive on the plot at the time of measurement (*VG_s_*) and *Dw*:
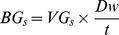



*VG_s_* was estimated by functions based on five years radial growth (determined from a tree core sample), *H* and height growth over five years [29].

Mean annual temperature and mean annual precipitations were assigned as climatic variables to each plot (temperature and precipitation, hereafter) based on available interpolated climatic maps for each country.

### Statistical analysis

First, for each forest type, we developed Generalized Linear Models to test for differences in wood production among tree species richness using the PROC-GENMOD procedure in SAS (version 9.2, SAS Institute Inc., Cary, NC, USA) with a normal error distribution and identity *link* function [30], and plot area as a covariate. When differences among tree species richness were significant, pair-wise differences of Least Square means (LS means) were tested. Likewise, we tested for differences in wood production among tree type richness.

To select the appropriate variables to be included in the Structural Equation Modeling (SEM) [31], we performed a stepwise regression analysis to test for the correlation of wood production with tree species richness, tree type richness, stand basal area, temperature and precipitation for each forest type, respectively.

The species richness-productivity relationship might vary with the spatial grain (i.e. plot size), the spatial extent (i.e. local, landscape, regional, continental or global), and also the ecological association scale (e.g. within or across community types) of the study [32]. Our forest surveys were conducted at local spatial scales, across a whole continent, and within 11 different forest types. Plot size ranged from 5 to 25 m radius, and was not always the same across forest inventories. Plot sampling areas were, however, within the size range considered appropriate for vegetation studies of European forests [33] and in forest inventories [28]. Therefore, our analysis captured tree alpha diversity across plots of similar size (Table 1). Following recommendations to investigate how the richness-productivity relationship changes across climatic gradients [34], we did not extrapolate the number of tree species to the regional scale but maintained the plot as the sample unit while the geographical extent was enlarged by incorporating plots from several countries. Values for tree species richness, stand basal area and wood production were standardized per unit sampling area prior to the analysis.

**Figure 1:**
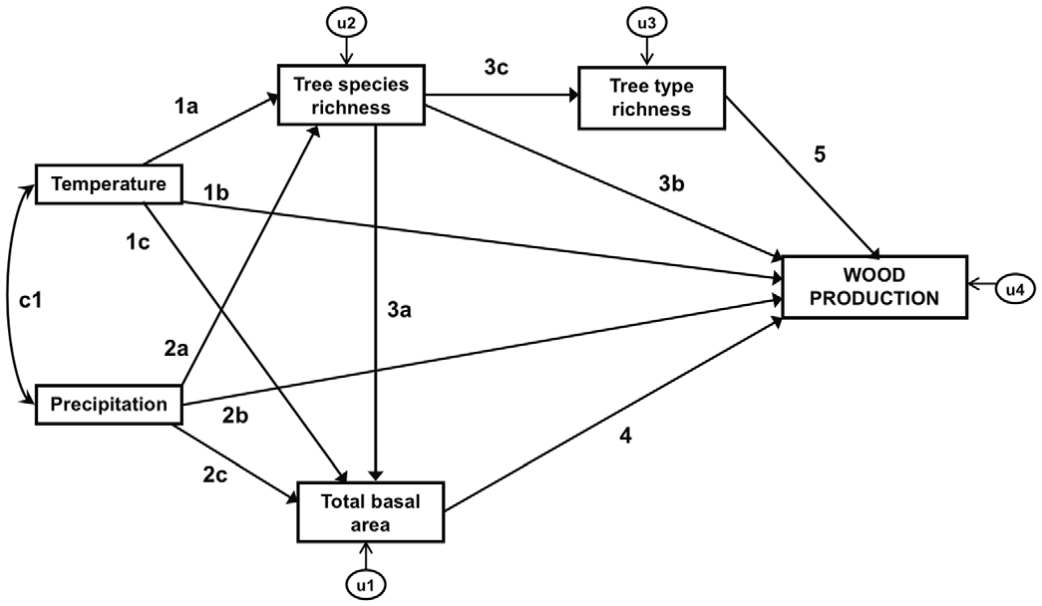
Structural Equation Model (SEM) for tree wood production. Single arrows represent causal paths (i.e. simple regressions between variables), whereas the double-headed arrow denotes correlation between mean annual precipitation and temperature. U_n_ values represent unexplained variance in each endogenous variance. The letters on each arrow indicate the standardized regression weights (path coefficients) between variables. Path coefficient values for each European forest type are given in Table 3.

Finally, SEM was used to test the above hypotheses. A SEM was constructed for each forest type. The model contains causal relationships among variables (Fig. 1), represented by single-headed arrows, and a correlational relationship between the two climatic variables that is represented by a double-headed arrow connecting temperature and precipitation. Direct effects of one variable on another are indicated by an arrow linking the two variables (e.g. tree species richness on wood production in Fig. 1), while indirect effects are those linked by an intermediate variable (e.g. tree species richness on wood production through tree type richness in Fig. 1) (see [35] for a detailed description of SEM procedures).

Due to large sample sizes in each forest type and the assumption of multivariate normality, standardized path coefficients were estimated using maximum likelihood techniques [35], [36]. We tested for both univariate and multivariate normality, applied transformations when necessary and examined for influential outliers (squared Mahalanobis distance, [37]). When normality assumptions were not met as a consequence of large sample sizes (i.e. alpine, broadleaved evergreen and coniferous Mediterranean forests), bootstrapping was used to evaluate statistical significance of each path coefficient [38], [39]. Subsequently, the goodness-of-fit was determined to test the degree to which the aprioristic SEM fits the sample data [40]. Since the commonly used chi-square test for the absolute model fit is sensitive to sample sizes and multivariate normality assumption of the input variables [40], the Comparative Fit Index (CFI) was used which does not depend on sample size as much as the chi-square test [41]. Values of CFI can range between 0 and 1, with values ≥0.90 confirming a good model fit.

For each forest type, we calculated the standardized regression coefficients associated with each path. These values represent the amount of change in one variable given a standard deviation unit change in the other one. We also calculated the coefficient of determination (R^2^) for each variable as an indication of the contribution of the model to the variation of that variable. The unexplained variance (u) of the model to each variable was also indicated (Table S2).

For models with CFI values ≥0.90, differences of path coefficients among forest types were determined through Multigroup analyses [36], [42]. SEM and Multigroup analyses were performed using the AMOS.18.0 software [38].

## Results

**Figure 2:**
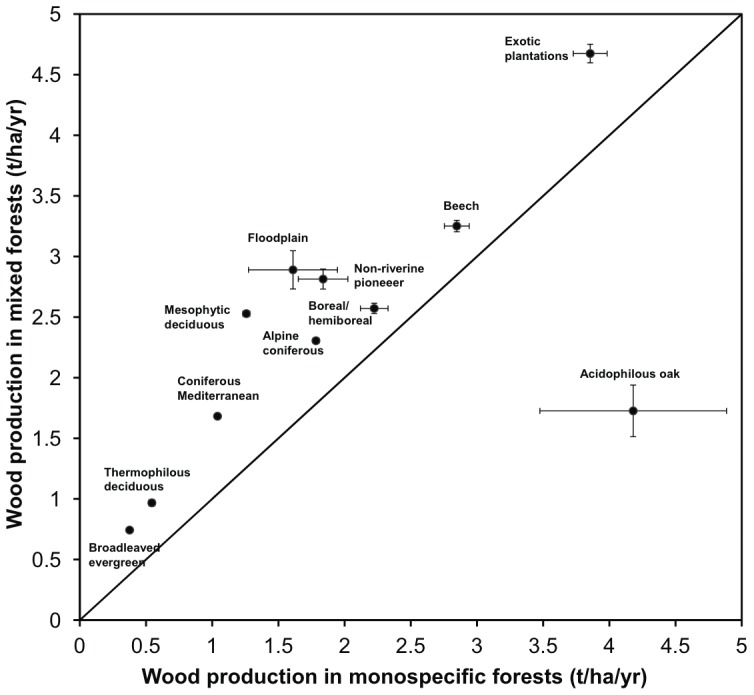
Tree wood production in pairs of monospecific and mixed forests. Values indicate means (±SE). Each point represents a different European forest type. The dashed line represents the line of unity.

Wood production was higher in mixed compared to monospecific forests of the same type as indicated by values falling above the line of unity in all forest types, except in acidophilous oak forests for which values were lower (Fig. 2). On average, wood production was 24.38% higher in mixed than in monospecific forests.

**Figure 3:**
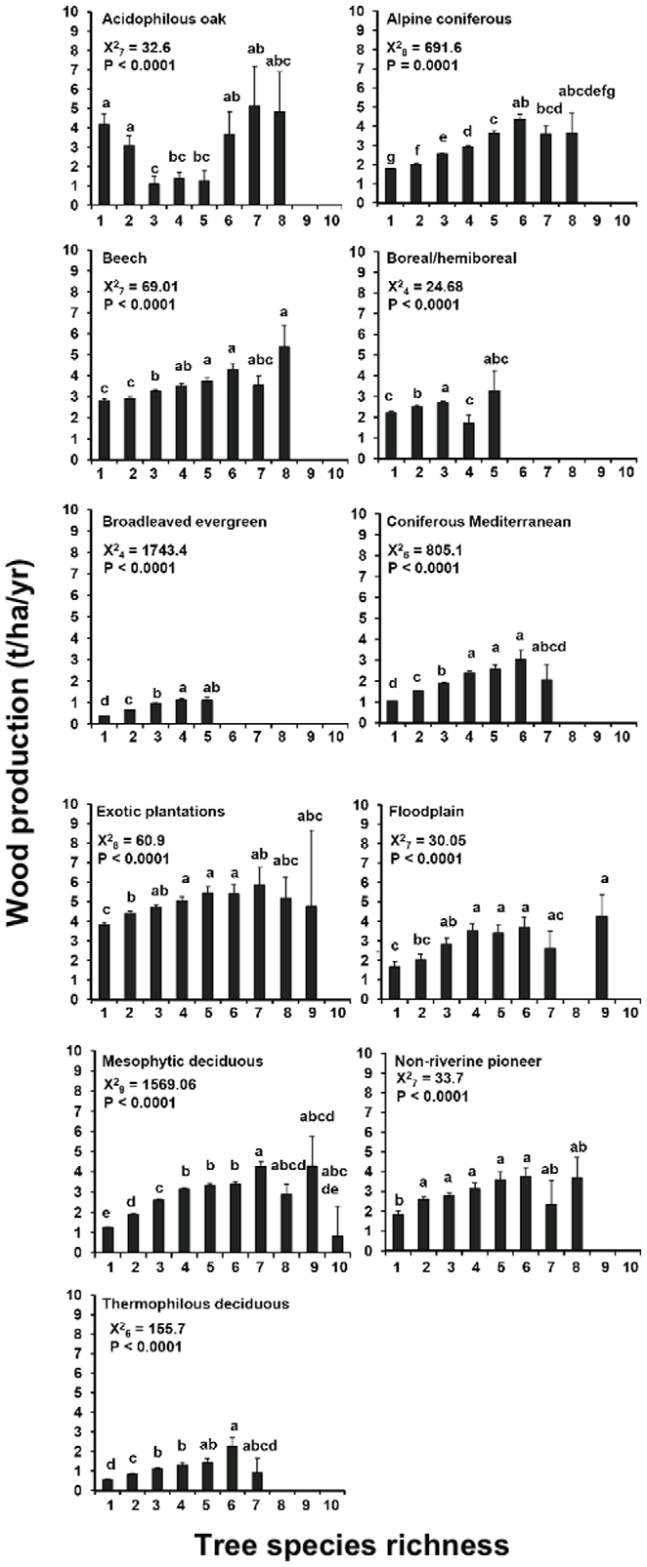
Tree wood production with increasing tree species richness. Values indicate LS means (±SE). Different letters above columns indicate significant differences between stands with different species richness according to GENMOD-procedure in SAS. n.s.  =  not significant.

**Figure 4:**
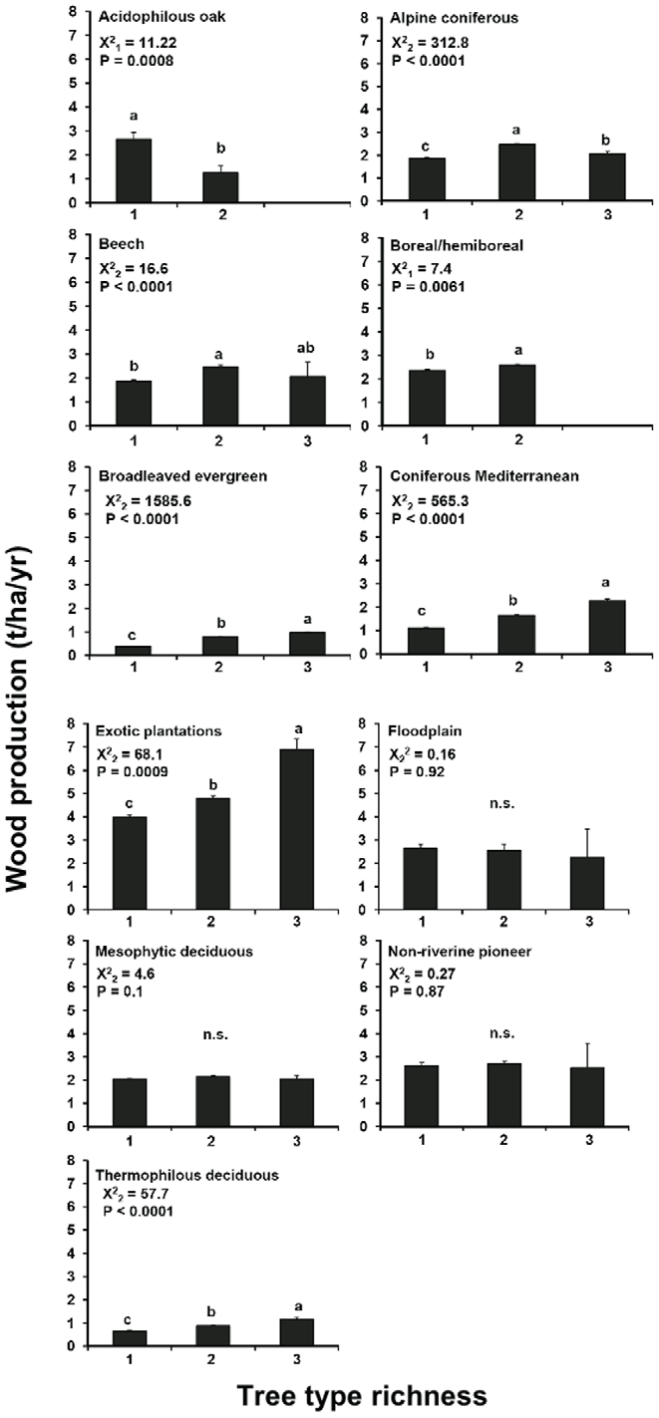
Tree wood production with increasing tree type richness. Values indicate LS means (±SE). Different letters above columns indicate significant differences between stands with different species richness according to GENMOD-procedure in SAS. n.s.  =  not significant.

Taken alone, wood production increased with tree species richness, at least from monospecific to mixed plots with 3–4 species, and then the relationship reached an asymptote (Fig. 3). In alpine forests, wood production increased up to six species, while in non-riverine pioneer forests maximum wood production was already reached in two species forests. In acidophilous forests, wood production decreased from monospecific to mixed plots with 3–5 species, while productivity in plots with 6–8 species was not significantly different from the monospecific ones. Similarly, wood production increased with tree type richness with the exception of floodplain, mesophytic deciduous and non-riverine pioneer forests, where the relationship was not significant, and acidophilous oak forests for which plots with only one tree type were more productive than with two tree types (Fig. 4). Alpine forests had a hump-shaped relationship, with two tree type forests being more productive than one and three tree type forests.

For each forest type, wood production was related to all variables tested in the stepwise analysis (Table 2). Stand basal area was the most important variable, explaining 54–84% of the variance in wood production. Overall, climatic variables were stronger determinants of wood production compared to tree species or tree type richness, and tree type richness explained more variance in wood production than tree species richness.

**Table 2:** Stepwise procedure on the relationship of abiotic and biotic variables with tree wood production.

	Abiotic variables		Biotic variables		
Forest types	Temperature	Precipitation	Stand basal area	Tree species richness	Tree type richness
Acidophilous oak	0.41	0.42	0.54	0.28	0.29
Alpine coniferous	0.51	0.61	0.81	0.43	0.54
Beech	0.69	0.69	0.81	0.48	0.65
Boreal and hemiboreal	0.54	0.59	0.62	0.53	0.54
Broadleaved evergreen	0.47	0.55	0.78	0.29	0.58
Coniferous Mediterranean	0.43	0.53	0.81	0.27	0.46
Exotic plantations	0.47	0.56	0.70	0.32	0.52
Floodplain	0.59	0.51	0.80	0.37	0.52
Mesophytic deciduous	0.57	0.64	0.84	0.58	0.53
Non-riverine pioneer	0.48	0.55	0.70	0.56	0.57
Thermophilous deciduous	0.48	0.54	0.78	0.20	0.50

For each forest type we indicate the adjusted R^2^ for each variable taken alone. All variables tested were also related to wood production across all 11 European forest types.

All the above variables were included in the SEM and were retained in the model. The CFI of the SEMs were ≥0.90 in all forest types except for broadleaved evergreen (0.89), non-riverine pioneer (0.78) and thermophilous deciduous (0.85) forests (Table S2). On average, 47% of the variance in wood production was explained by the model, with highest values in coniferous Mediterranean forests (68%), and alpine coniferous and mesophytic deciduous (>55%); and lowest values (19%) in boreal and hemiboreal forests (Table S2).

Tree species richness had a low direct effect on wood production (path 3b, Table 3). However, in almost all forest types, stand basal area increased with tree species richness (path 3a, Table 3), and stand basal area was the variable with the largest positive effect on wood production (path 4, Table 3). Therefore, the effect of tree species richness on wood production is mainly indirect by increasing stand basal area. Tree type richness increased wood production in some forest types, namely alpine coniferous, coniferous Mediterranean, broadleaved evergreen and exotic plantations. However, path coefficients were small (path 5, Table 3) and of a similar magnitude to tree species richness.

**Table 3:** Structural equation modelling (SEM) path coefficients.

	Path coefficients																							
	1a		1b		1c		2a		2b		2c		3a		3b		3c		4		5		c1	
Acidophilous oak	0.77	***	−0.09	ns	0.00	ns	−0.37	***	−0.03	ns	0.22	*	0.00	ns	−0.15	ns	0.23	*	0.48	***	−0.11	ns	0.53	***
Alpine coniferous^1^	0.06	***	0.09	***	−0.17	***	0.47	***	0.12	***	0.19	***	0.11	***	0.00	ns	0.47	***	0.72	***	0.03	***	−0.43	***
Beech	−0.04	*	0.21	***	−0.06	**	0.33	***	0.14	***	0.12	***	0.10	***	−0.02	ns	0.41	***	0.64	***	0.03	ns	−0.4	***
Boreal and hemiboreal	−0.08	**	0.27	***	0.35	***	−0.05	*	−0.03	ns	−0.02	ns	0.18	***	0.04	ns	0.65	***	0.27	***	0.00	ns	0.61	***
Broadleaved evergreen^1^	−0.39	***	−0.06	***	−0.27	***	0.03	***	0.05	***	0.34	***	−0.05	***	0.06	***	0.35	***	0.7	***	0.10	***	0.11	***
Coniferous Mediterranean^1^	−0.06	***	0.03	***	−0.1	***	0.14	***	0.19	***	0.25	***	0.48	***	0.01	ns	−0.07	***	0.76	***	0.03	***	−0.15	***
Exotic plantations	−0.58	***	0.24	***	−0.19	***	−0.19	***	0.15	***	0.3	***	0.06	***	−0.13	***	0.39	***	0.71	***	0.06	***	−0.65	***
Floodplain	−0.39	***	0.13	*	0.04	ns	0.28	***	0.017	ns	−0.08	ns	0.14	*	0.05	ns	0.25	**	0.71	***	−0.11	*	−0.32	***
Mesophytic deciduous	−0.13	***	0.043	***	−0.06	***	0.31	***	0.1	***	0.2	***	0.25	***	0.08	***	0.24	***	0.7	***	−0.07	***	−0.36	***
Non-riverine pioneer	−0.22	***	0.17	***	0.22	***	0.1	*	−0.07	ns	0.00	ns	0.33	***	0.03	ns	0.27	***	0.51	***	0.05	ns	0.69	***
Thermophilous deciduous	−0.18	***	−0.03	ns	−0.1	***	−0.24	***	0.13	***	0.3	***	−0.07	*	0.10	*	0.28	***	0.69	**	−0.03	ns	0.16	***

For each forest type we indicate the standardized regression weights of the paths according to the nomenclature indicated in Figure 1.

1 *Forest data was analyzed through bootstrapping*. Significance of the path coefficients: *P<0.05, ** P<0.005, ***P<0.0001, ns  =  not significant.

Temperature increased wood production in most forest types (path 1b, Table 3). On the contrary, temperature had almost always a negative effect on tree species richness except in acidophilous and alpine coniferous forests where it was positive (path 1a, Table 3). Precipitation increased wood production in most forests, except in acidophilous oak, boreal and hemiboreal, floodplain and non-riverine pioneer forests where the relationship was not significant (path 2b, Table 3). Precipitation also increased species richness, except in acidophilous oak, boreal and hemiboreal, exotic plantations and thermophilous deciduous forests where it was negative (path 2a, Table 3).

Not only was the direction of the relationship between climatic variables and wood production different compared to that of species richness, it also differed in magnitude. That is, even within a forest type the effect of climate on tree species richness and wood production could be in opposite directions, be significant for one variable and not significant for the other, or of different magnitude. For example, in acidophilous oak forests, temperature and precipitation had a non-significant effect on wood production, but temperature increased tree species richness (77% of the variation explained) while precipitation affected tree species richness negatively (37% of the variation explained). Multigroup analyses revealed that path coefficients among forest types were significantly different (Table S3). However, differences between forest types were dependent on the path under consideration (Table S4).

## Discussion

We found a positive relationship between tree richness and wood production in most European forest types. Our analysis is the first to describe this relationship at the local scale for the largest dataset across a continent, encompassing a wide range of climatic conditions. This result is in line with other regional studies showing higher productivity in mixed compared to monospecific forests [43], [44]. We found European mixed forests to be on average 24% more productive than monospecific forests. Although we do not have precise information on the management history of these forests, most of our study plots were not plantations but natural forests. Moreover, even if some might be plantations they had not been managed during the inventory measurement periods. This indicates that the positive relationship between species richness and productivity is found in structurally complex woody systems, encompassing a wide range of environmental conditions [45].

As also found in other ecosystems, in many forest types maximum wood production was reached at medium levels of species richness. There may be several non-exclusive explanations for this pattern. Functional redundancy and niche overlap may occur at high levels of species richness [2]. Therefore, a complete exploitation of available resources for wood production seems to be reached faster in high compared to low species rich forests. Alternatively, the saturation of the tree species richness-productivity relationship may be a consequence of higher levels of evenness in plots of low (i.e. 2–3 species) compared to high tree species richness. Tree species evenness has been found to be a better predictor of wood production than tree species richness [44]. Furthermore, plots of high tree richness are less common than plots of low richness [12], [13]. Plots of high richness are therefore more variable in wood production due to small sample sizes, but possibly also due to a larger variation in species composition and a lower abundance of rare species.

The positive association between tree species richness and wood production was mediated by an increase in tree stand basal area with species richness. Although stand age was not available, most European forests have an uneven-aged structure, have been highly managed historically, and are at an early seral stage [25]. In these circumstances, stand basal area has not reached its maximum yet [46] and tree species richness is high [47]. Although our study cannot elucidate the ecological mechanisms underlying the positive relationship between tree species richness and wood production, two non-mutually exclusive mechanisms have been hypothesized to drive this observation: the complementarity effect and the sampling effect. The first hypothesis postulates that species rich stands are most efficient in resource use because they contain species with a diverse array of ecological traits such as multilayered canopies or roots at different depths that optimize ecosystem resource use. Complementarity can result from niche partitioning and/or facilitation among species with different traits, decreasing competition in diverse communities [20], [48]. Alternatively, the positive association might be explained by a sampling effect, whereby species rich stands are more likely to contain and become dominated by at least one species highly efficient in resource use that accounts for most of the production in the community [1], [49]. Both mechanisms can act simultaneously or there might be transitions between them over large time spans [6]. Moreover, their importance might depend on the forest type. For example, in climatically stressful Mediterranean conditions, mixed forests containing species of low productivity might achieve higher wood production because of species niche partitioning in water use [12]. On the other hand, in many European forests, traditional management has favored economically important species and highly productive varieties (e.g. exotic trees). When abandoned and colonized with other tree species, these stands might still remain highly productive because of the sampling effect of highly productive trees. Long term experimental tree plantations are needed to test the mechanisms underlying the positive signal between tree species richness and wood production and how it might change over time [50].

The positive relationship between tree species richness and wood production mediated by an increase in basal area remained significant when climatic factors were included in the models. This indicates that climatic differences are not the sole explanation for differences in wood production along a gradient of species richness. Moreover, our analysis shows that the influence of temperature and precipitation has on wood production are highly dependent on forest type. Our analyses also reveal that climate does not influence wood production and tree species richness in parallel [27].

In more than half of the forest types, wood production was positively related to tree type richness. However, often there were no significant differences between two- and three-tree type mixtures. In some forest types, the relationship was not significant, negative, or hump-shaped. This idiosyncrasy was unexpected as we had predicted tree type richness to be functionally as relevant as species richness. The low number of tree types in European forests (i.e. evergreen conifers, deciduous conifers, evergreen broadleaved and deciduous broadleaved) is possibly the cause of these inconsistencies among forest types. Moreover, tree types are possibly too coarse to underpin differences in functional traits responsible for wood production. Tree species richness might better reflect functional trait diversity than the tree type richness used in our study. Biodiversity categories based on growth forms are “soft traits” that may mask within-group variability of traits [51]. Recent studies have shown that functional diversity indices based on traits relating to reproduction, growth, successional status and resource use perform better than indices of species diversity [13], [52]. However, due to the large variation in species composition in European forest inventories, there is still not enough information on functional species traits for many species, especially Mediterranean and alpine tree species.

Overall, our study shows for the first time across a continent that local tree wood production is positively associated with local tree species richness in many forest types, even when controlling for climatic variation. Although wood production is just one process of the global C cycle, tree growth is the principal forest C flux contributing to atmospheric CO_2_ sequestration by the biosphere [53]. Our results suggest that preserving forests with a high alpha diversity could substantially increase C sequestration at the local scale by increasing wood production. Thus, forest related biodiversity issues, although neglected until now, should be incorporated in management and policy plans for C sequestration.

## Supporting Information

Table S1 **Main characteristics of the forest inventories.**
(DOC)

Table S2 **Results of the structural equation model (SEM).**
(DOC)

Table S3 **Goodness-of-fit statistics for multigroup analyses.**
(DOC)

Table S4 **Summary of multigroup comparison among forest types for single path coefficients.**
(DOC)
