# Supplementary material for: Disentangling Biodiversity and Climatic Determinants of Wood Production
Source: PLoS One. 2013 Feb 20;8(2):e53530. doi: 10.1371/journal.pone.0053530 (PMC3577818; doi:10.1371/journal.pone.0053530)
Supplement: Table S1 — Main characteristics of the forest inventories. (DOC) [file pone.0053530.s001.doc]

**Supporting information**

**Table S1.** Main characteristics of the forest inventories from five European countries. Grid area indicates the forested area of reference from which the plot was sampled.

**Country Nº plots Sampling years Time interval Minimum tree DBH Plot size Grid area References**

**(yrs) (cm) (m^2^) (m)**

**France** 14070 1995-2001 5 7.5 359 500 x 500 Kunstler et al. 2011

Chave et al. 2009

Robert et al. 2010

**Netherlands** 422 1983-2000 8 5 499 50 x 50 Jansen et al. 1996

Broekmeyer 1999

Totkamp et al. 2006

Bijlsma 2008

**Spain** 37133 1989/90-2000/01 6.6-12.5 7.5 79, 200, 1000 x 1000 Alberdi et al. 2010

707, 1964 Vilà et al. 2008

**Sweden** 3281 2003/4-2008/9 5 4-10 314 300 x 1200 Axelsson et al. 2010

WSL 2010

**Switzerland** 359 1993/4-2008/9 8.9-13.5 12 200 1400 x 1400 Brändli 2010

Lanz et al. 2

**References**

Alberdi I, Condés S, Martínez J, Saura S, Sánchez G, et al. (2010) Spain. In: Tomppo E, Gschwantner T, Lawrence M, McRoberts RE, editors. National Forest Inventories - Pathways for Common Reporting. Springer, Heidelberg, Dordrecht, London, New York. pp. 527-540.

Axelsson AL, Ståhl G, Söderberg U, Peterson H, Fridman J, et al, ( 2010). Sweden. In: Tomppo E, Gschwantner T, Lawrence M, McRoberts RE, editors. National Forest Inventories - Pathways for Common Reporting. Springer, Heidelberg, Dordrecht, London, New York. pp. 541-553.

Bijlsma RJ (2008) Bosreservaten: koplopers in de natuurlijke ontwikkeling van het Nederlandse boslandschap. In Dutch: [Forest reserves: leaders in the natural development of the Dutch forested landscape]. Alterra-rapport 1680. pp. 50.

Brändli UB (Réd.) (2010) Inventaire forestier national suisse. Résultats du troisième inventaire 2004-2006. Birmensdorf, Institut fédéral de recherches sur la forêt, la neige et le paysage WSL. Berne, Office fédéral de l'environnement, OFEV. In French: [Swiss National Forest Inventory. Results of the third survey 2004-2006. Birmensdorf, Swiss Federal Institute for Forest, Snow and Landscape Research WSL. Bern, Federal Office for the Environment, FOEN]. pp. 312.

Broekmeyer M (1999). The Netherlands. In: Parviainen J, Little D, Doyle M, O’Sullivan A, Lettunen M, Korhonen M, editors . Research in Forest Reserves and Natural Forests in European Countries. Country Reports for the Cost Action E4: Forest Reserves Research Network. EFI Proceedings No.16. pp. 177-194.

Chave J, Coomes D, Jansen S, Lewis SL, Swenson NG, et al. (2009) Towards a worldwide wood economics spectrum. Ecol Lett 12: 351-366.

Jansen JJ, Sevenster J, Faber PJ (1996) Opbrengsttabellen voor belangrijke boomsoorten in Nederland. In Dutch: [Yield tables for important tree species in the Netherlands]. Wageningen, IBN-DLO. IBN rapport 221.

Kunstler G, Albert C, Courbaud B, Lavergne S, Thuiller W, et al. (2011) Hierarchical bayesian analyses of tree-tree interactions reveal that competition varies in importance but not in intensity along bioclimatic gradients. J Ecol 29: 1247-1257.

Lanz A, Brändli UB.,Brassel P, Ginzler C, Kaufmann E, et al. (2010) Switzerland. In: Tomppo E, Gschwantner T, Lawrence M, McRoberts RE, editors. National Forest Inventories - Pathways for Common Reporting. Springer, Heidelberg, Dordrecht, London, New York. pp. 555-565.

Robert N, Vidal C, Colin A, Hervé JC, Hamza N, et al. (2010) France. In: Tomppo E, Gschwantner T, Lawrence M, McRoberts RE, editors. National Forest Inventories - Pathways for Common Reporting. Springer, Heidelberg, Dordrecht, London, New York, pp. 207–221.

Tolkamp GW, Van Den Berg CA, Nabuurs GJMM, Olsthoorn AFM (2006) Kwantificering van beschikbare biomassa voor bio-energie uit Staatsbosbeheerterreinen. In Dutch: [Quantification of available biomass for bio-energy from the terrains of the State Forest Service] Wageningen, Alterra. Alterra-rapport 1380, pp. 46.

Vayreda J, Martínez-Vilalta J, Gracia M, Retana J (2012) Forest structure and management interact with recent changes in climate to determine the tree carbon stock change in peninsular Spain forests. Glob Chang Biol 18: 1028–1041.

Vilà M, Vayreda J, Comas Ll, Ibáñez JJ, Mata T, et al. (2007) Species richness and wood production: a positive association in Mediterranean forests. Ecol Lett 10: 241-250.

WSL (2010) Schweizerisches Landesforstinventar LFI. Daten der Erhebungen 1993-1995 und 2004-2006. 280510UU. Eidg. Forschungsanstalt WSL, In German: [ Swiss National Forest Inventory (LFI). Data of the surveys of 1993-1995 and 2004-2006. Contract 280510UU, Swiss Federal Institute WSL], Birmensdorf.
