# Supplementary material for: Disentangling Biodiversity and Climatic Determinants of Wood Production
Source: PLoS One. 2013 Feb 20;8(2):e53530. doi: 10.1371/journal.pone.0053530 (PMC3577818; doi:10.1371/journal.pone.0053530)
Supplement: Table S2 — Results of the structural equation model (SEM). (DOC) [file pone.0053530.s002.doc]

**Supporting information**

**Table S2.** Results of the structural equation model (SEM) on the effects of forest structure and climatic variables on tree wood production in 11 European forest types. For each forest type we indicate the model fit parameters (CFI), coefficients of determination (R^2^) and unexplained variance (u) for stand basal area (BAT), tree species richness (RICH), tree type richness (TTR) and wood production (WPR).

|  | **Model fit** | **R^2^** | | | | **u1** | **u2** | **u3** | **u4** |
| --- | --- | --- | --- | --- | --- | --- | --- | --- | --- |
|  | **CFI** | **BAT** | **RICH** | **TTR** | **WPR** | **BAT** | **RICH** | **TTR** | **WPR** |
| **Acidophilous oak** | 0.91 | 0.05 | 0.42 | 0.06 | 0.29 | 0.97 | 0.76 | 0.97 | 0.84 |
| **Alpine coniferous^1^** | 0.96 | 0.14 | 0.20 | 0.22 | 0.57 | 0.93 | 0.89 | 0.88 | 0.66 |
| **Beech** | 0.97 | 0.05 | 0.12 | 0.17 | 0.45 | 0.98 | 0.94 | 0.91 | 0.74 |
| **Boreal and hemiboreal** | 0.99 | 0.13 | 0.02 | 0.43 | 0.19 | 0.93 | 0.99 | 0.76 | 0.90 |
| **Broadleaved evergreen^1^** | 0.89 | 0.16 | 0.15 | 0.12 | 0.55 | 0.92 | 0.92 | 0.94 | 0.67 |
| **Coniferous Mediterranean^1^** | 0.95 | 0.08 | 0.03 | 0.23 | 0.68 | 0.96 | 0.99 | 0.88 | 0.56 |
| **Exotic plantations** | 0.95 | 0.22 | 0.23 | 0.15 | 0.49 | 0.88 | 0.88 | 0.92 | 0.71 |
| **Floodplain** | 1.00 | 0.05 | 0.30 | 0.02 | 0.53 | 0.98 | 0.83 | 0.99 | 0.69 |
| **Mesophytic deciduous** | 0.97 | 0.15 | 0.15 | 0.06 | 0.56 | 0.92 | 0.92 | 0.97 | 0.66 |
| **Non-riverine pioneer** | 0.78 | 0.14 | 0.03 | 0.07 | 0.31 | 0.93 | 0.99 | 0.96 | 0.83 |
| **Thermophilous deciduous** | 0.85 | 0.10 | 0.11 | 0.08 | 0.53 | 0.95 | 0.95 | 0.96 | 0.69 |
| ***^1^*** *Forest data was analyzed through bootstrapping* | | | |  |  |  |  |  |  |
