# Supplementary material for: Disentangling Biodiversity and Climatic Determinants of Wood Production
Source: PLoS One. 2013 Feb 20;8(2):e53530. doi: 10.1371/journal.pone.0053530 (PMC3577818; doi:10.1371/journal.pone.0053530)
Supplement: Table S3 — Goodness-of-fit statistics for multigroup analyses. (DOC) [file pone.0053530.s003.doc]

**Supporting information**

**Table S3.** Goodness-of- fit statistics for multigroup analyses. The first row (None) indicates the maximum likelihood X^2^ estimates (ML_Χ_^2^) from constraining all free parameters to the same value among forest types. The following rows are the effect on X^2^ of realizing each single free parameter on each time. ∆ML_Χ_^2^ shows differences in Χ^2^ between the constraint model and the rest of models; probability of ∆ML_Χ_^2^ represents the probability that the release of that parameter improves the model significantly. Patch coefficient nomenclature is indicated in Fig. 1.

| **Free parameters for which between-group equality constraint was released** | **ML_Χ_^2^** | **∆ML_Χ_^2^** | **Probability of ∆ML_Χ_^2^** |
| --- | --- | --- | --- |
| **None** | 54710.87 |  |  |
| **Free path 1a** | 45408.62 | 9302.25 | ******* |
| **Free path 1b** | 35269.93 | 19440.94 | ******* |
| **Free path 1c** | 52019.26 | 2691.61 | ******* |
| **Free path 2a** | 44534.50 | 10176.37 | ******* |
| **Free path 2b** | 35055.31 | 19655.56 | ******* |
| **Free path 2c** | 52455.73 | 2255.13 | ******* |
| **Free path 3a** | 52316.45 | 2394.42 | ******* |
| **Free path 3b** | 35524.94 | 19185.93 | ******* |
| **Free path 3c** | 51578.86 | 3132.01 | ******* |
| **Free path 4** | 35229.96 | 19480.91 | ******* |
| **Free path 5** | 35598.17 | 19112.70 | ******* |
| **Free path C1** | 52587.09 | 2123.78 | ******* |
