# Supplementary material for: Disentangling Biodiversity and Climatic Determinants of Wood Production
Source: PLoS One. 2013 Feb 20;8(2):e53530. doi: 10.1371/journal.pone.0053530 (PMC3577818; doi:10.1371/journal.pone.0053530)
Supplement: Table S4 — Summary of multigroup comparison among forest types for single path coefficients. (DOC) [file pone.0053530.s004.doc]

**Supporting information**

**Table S4.** Summary of multigroup comparison among forest types for single path coefficients. Values represent Critical ratios (CR) for differences between parameters among forest types. If |CR| < 1.96, the test is statistically non-significant (α ≤ 0.05) indicating that the parameter compared is equal between forest types. In bold we indicate significant differences between forest types for each coefficient path compared.
